# Malignant adenomyoepithelioma of the breast: A rare case report

**DOI:** 10.1016/j.ijscr.2019.04.045

**Published:** 2019-05-07

**Authors:** Mi Jin Kim, Cheol Seung Kim, Myoug Jin Ju, Young Sam Park

**Affiliations:** a Department of General Surgery, Presbyterian Medical Center, Republic of Korea; b Department of Pathology, Presbyterian Medical Center, Republic of Korea

**Keywords:** Breast neoplasms, Adenomyoepithelioma, Myoepithelial tumor, Breast cancer, Malignant neoplasm of breast

## Abstract

**Introduction:**

Adenomyoepithelioma of the breast is a rarely reported and mostly benign disease that seldom undergoes malignant transformation.

**Presentation of the case:**

Here, we present a case of malignant adenomyoepithelioma of the breast in a patient who initially presented with pain following an excision procedure at local clinic. The condition was finally diagnosed after a third surgical procedure, pathologic analysis with hematoxylin and eosin staining, and immunohistochemistry analysis to detect smooth muscle actin and S100 expression.

**Conclusion:**

The patient developed no complications or recurrences after a total mastectomy with sentinel node dissection.

## Highlights

• Adenomyoepithelioma is an uncommon benign disease that occurs in the breast.
• Although not common, adenomyoepithelioma is associated with metastasis with malignant changes.
• Total mastectomy is known to be the best treatment method, and the effect of chemotherapy or radiation therapy is not known clearly.

## 1 Introduction

Adenomyoepithelioma is as very rare type of benign tumor of the breast. These lesions rarely undergo a malignant transformation and are even less likely to metastasize. However, malignant adenomyoepithelioma is difficult to differentiate from other benign diseases such as intraductal papilloma, tubular adenoma, and sclerosing adenosis. In this report, we describe our experience with a patient who underwent a mastectomy with sentinel node dissection and was diagnosed with malignant adenomyoepithelioma of the breast following repeated surgeries for the treatment of recurrent breast tumors.

This study was approved by the appropriate Ethical Review Board committee of our institution. Research was conducted in accordance with the 1964 Declaration of Helsinki and its later amendment.

This study was reported according to the SCARE guidelines [1].

## 2 Presentation of case

A 56-year-old woman presented with mastalgia after a mass excision from the right breast at another hospital 1 month earlier. At that time, she had received a histopathological diagnosis of adenomyoepithelioma. She was referred to our hospital because of continuous postoperative pain. A physical examination revealed surgical wound tenderness with no palpable mass in the breast. An ultrasound scan revealed multiple hypoechoic mass-like lesions indicative of either a simple postsurgical hematoma or a tumor. We therefore suggested a wait-and-see policy for 3 months, by which time the lesion had become smaller but had not resolved and the patient continued to complain of pain.

Next, we aspirated the lesion and withdrew a volume of 5 cc, which contained only inflammatory cells and no tumor cells. As the patient's pain decreased after this procedure, we again decided upon a wait-and-see policy. However, after 3 months, she returned to the clinic complaining of mastalgia at the surgical excision site. This time, an ultrasound revealed larger and more numerous breast tumor-like lesions. The patient underwent a wide excision, and a histopathological examination revealed intraductal papilloma with myoepithelial cell hyperplasia. An immunohistochemistry analysis indicated positivity for CD10, P63, and E-cadherin.

Although the patient reported no complaints after the procedure, a follow-up ultrasound scan conducted 6 months postoperatively revealed a recurrence of multiple tumor-like lesions, of which the largest was 1.5 cm in size. A core needle biopsy yielded epithelial–myoepithelial cells indicative of adenomyoepithelioma or intraductal papilloma. A third wide excision surgery was performed to remove the masses. Subsequently, a pathological diagnosis of malignant adenomyoepithelioma with 10/10 high-power field (HPF) mitosis and 5% necrosis was made. An immunohistochemical examination detected positive P63, smooth muscle actin, and S-100 expression in the myoepithelial cells and cytokeratin (CK) 7 and CK5 expression in the epithelial cells.

**Fig. 1:**
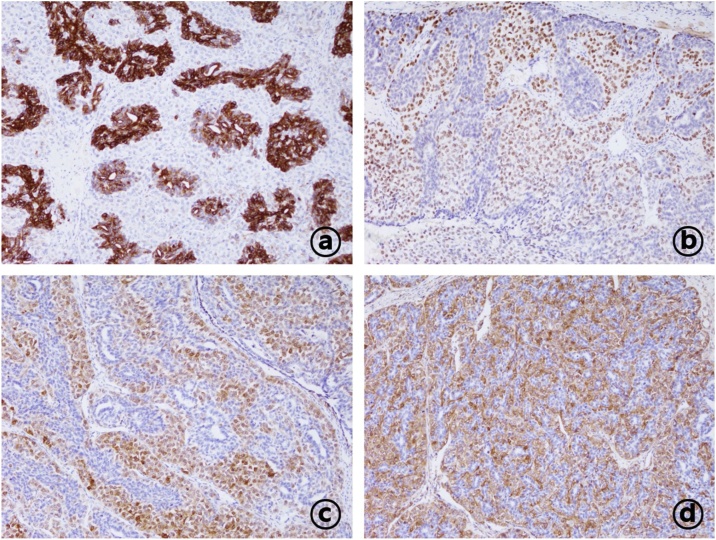
Immunohistochemical staining of the resected tissue (100× magnification). (a) Epithelial cells were very strongly positive for cytokeratin-7 (CK7). (b) Nuclear P63 staining in the myoepithelial component confirmed the differentiation of the myoepithelium. (c) Strong nuclear S-100 staining confirms cell proliferation in the myoepithelial component of the malignant adenomyoepithelioma. (d) Positive smooth muscle actin staining in myoepithelial cells.

**Fig. 2:**
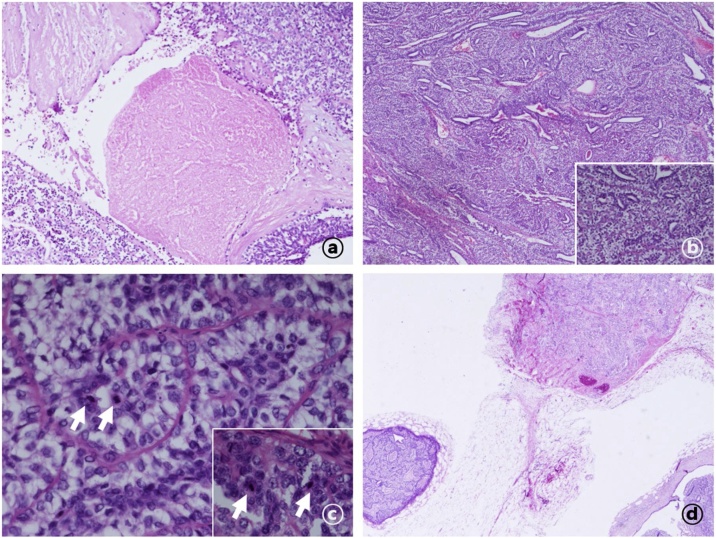
Pathologic findings in an adenomyoepithelioma (hematoxylin and eosin stain). (a) Necrotic changes in the malignant adenomyoepithelioma (10× magnification). (b) Tubular architecture in a malignant adenomyoepithelioma (40×). (c) Inner epithelial and outer myoepithelial layer with clear cytoplasm (400×). The white arrows indicate high levels of mitotic activity. (d) Adenomyoepithelioma with a satellite nodule.

Estrogen receptor and progesteron receptor detected negative, and c-erb2 shown weakly positive (1+). (Fig. 1, Fig. 2). Because the resection margin exhibited tumor involvement, we conducted a whole-body bone scan and positron emission tomography–computed tomography scan to check for distant metastases, which yielded negative results. Accordingly, we performed a total mastectomy with sentinel node dissection rather than breast-conservation surgery according to the wishes of the patient and her family. The resection margins were evaluated to confirm the absence of tumor. The nodes were negative for metastasis. Postoperatively, the patient did not receive any adjuvant systemic therapy and has remained disease-free for 2 years according to follow-up ultrasound and magnetic resonance imaging.

## 3 Discussion

Adenomyoepithelioma of the breast was first described as a tumor with simultaneously proliferating epithelial and myoepithelial cells by Hanperl in 1970. Since then, this disease has been reported in patients ranging in age from 22 to 92 years, with a mean age of onset of approximately 60 years. The most common complaint associated with this tumor type is a self-palpable solitary nodule. In contrast to the complaint of persistent breast lesion pain in the present case, pain and nipple discharge are rarely reported in the literature [[2], [3], [4], [5]].

The pathologic features of adenomyoepithelioma have been well described and generally include a firm-to-hard nodular mass, although lesions with a soft texture or ill-defined margins have been described. These tumors range in size from 1 to 17 cm, may contain small cystic areas, and can be classified into three variants: spindle cell, lobulated, and tubular type; the last variant is most common [6,7]. The immunohistochemical analysis of an adenomyoepithelioma generally yields positive staining for AE1/AE3, epithelial membrane antigen, low-molecular-weight keratin, CK antibody, CK AE1/3, CK CAM 5.2, and/or CK7 in the luminal surfaces of glandular cells in the epithelial component, as well as p53, smooth muscle myosin heavy chain (most sensitive), CK5, CD10, calponin, actin, S100, and Ki-67 in the myoepithelial component [2,3,6,7]. Consistent with previous findings, the immunohistochemistry analysis in the present case yielded positive staining for smooth muscle actin and S-100 in myoepithelial cells, which facilitated the diagnosis of AME.

The differential diagnosis of adenomyoepithelioma includes intraductal papilloma, nipple adenoma, clear cell carcinoma, metaplastic tumors associated with papilloma, invasive ductal cancer with necrosis or hemorrhage, tubular adenoma, sclerosing adenosis, complex sclerosing lesion, ductal adenoma, low-grade adenosquamous carcinoma, metaplastic carcinoma, malignant myoepithelioma, and papillary carcinoma [2,3,7,8]. Characteristically, adenomyoepitheliomas tend to exhibit benign clinical behavior, although malignant transformation has been reported in a small number of cases. This transformation is indicated by features such as prominent cytological atypia, high mitotic activity, necrosis, and an infiltrative growth pattern [6,7,9]. Generally, only one cellular component (epithelial or myoepithelial) becomes malignant, whereas the presence of malignant changes in both cell types is an extremely rare occurrence [10,11].

The prognosis of metastatic malignant adenomyoepithelioma is poor. Distant metastases of malignant adenomyoepithelioma to the lung, brain, thyroid, and chest wall have been reported [5]. By contrast, metastasis to the axillary lymph node is extremely rare and has been reported only in one case [5,12]. This pattern suggests that metastatic malignant adenomyoepithelioma spreads mainly through the hematogenous route, rather than the lymphatic system [3,5,7,9,[12], [13], [14]]. The metastatic potential appears to depend on the grade of the malignant component, which is determined by the number of mitoses in the tissue. Up to 6/10 HPF is characteristic of aggressive adenomyoepithelioma, whereas a metastatic lesion will contain more than 8/10 HPF [7].

The proper surgical treatment of adenomyoepithelioma involves a wide excision with a negative resection margin. Re-excision via simple mastectomy or extended resection is recommended for cases with a narrow or incomplete excision margin. As metastasis to the axillary lymph node is rare, some authors recommend simple mastectomy with sentinel lymph node biopsy as the treatment of choice. Other studies have suggested adjuvant chemotherapy or radiation therapy, although the outcomes have not been favorable [3,5,7,11,15,16].

## 4 Conclusion

Malignant adenomyoepithelioma, a rare malignant neoplasm of the breast, is possible to multiple metastases. For that reason, treatment of recurrent benign tumors in breast should be cautious.

## Conflicts of interest

The researcher claims no conflicts of interest.

## Sources of funding

The author(s) received no specific funding for this work.

## Ethical approval

This study was approved by the appropriate Ethical Review Board committee of our institution. Research was conducted in accordance with the 1964 Declaration of Helsinki and its later amendment.

## Consent

Written informed consent was obtained from the patient for publication of this case report and accompanying images. A copy of the written consent is available for review by the Editor-in-Chief of this journal on request.

## Author contribution

Study concept or design: Cheol Seung Kim, young Sam Park.

Data collection: Mi Jin Kim, Myoung Jin Ju.

Data analysis or interpretation: Mi Jin Kim.

Writing the paper: Mi Jin Kim.

## Registration of research studies

This is not a clinical trial, but a case report

## Guarantor

Mi Jin Kim.

## Provenance and peer review

Not commissioned, externally peer-reviewed.
